# Supplementary material for: Single-neuron representation of learned complex sounds in the auditory cortex
Source: Nat Commun. 2020 Aug 31;11:4361. doi: 10.1038/s41467-020-18142-z (PMC7459331; doi:10.1038/s41467-020-18142-z)
Supplement: Supplementary file 1 — Supplementary Information [file 41467_2020_18142_MOESM1_ESM.pdf]

Supplementary Information

Single-neuron representation of learned complex sounds in the auditory cortex

Wang et al.

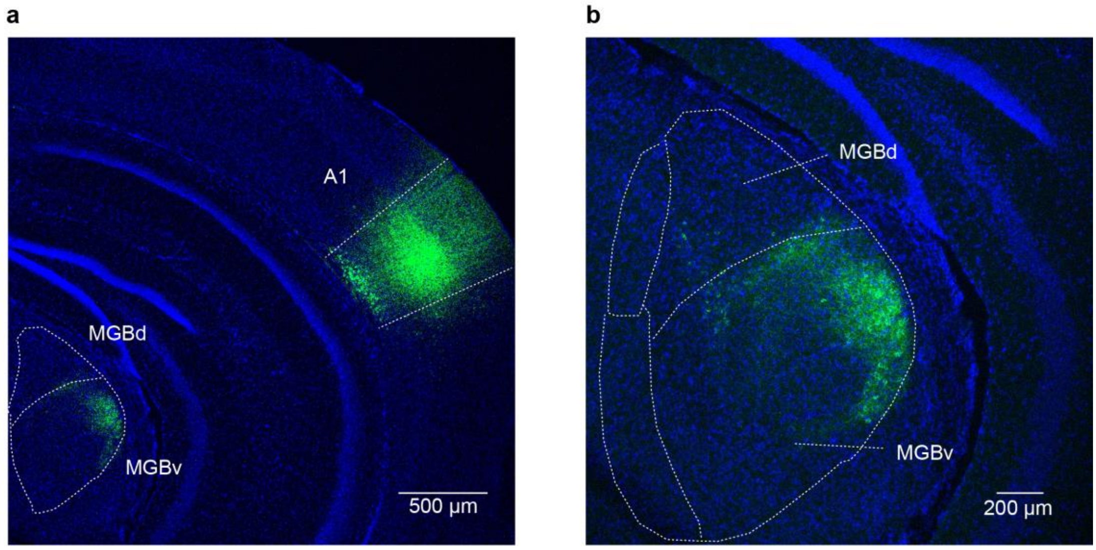

Supplementary Fig. 1 Retrograde labelling of MGBv to verify the location of A1 in mice. a A coronal brain slice shows the injection site of the retrograde neuronal tracer CTB-green (in A1) of an example mouse. **b** Enlarged view of the medial geniculate body showing its ventral (MGBv) and dorsal (MGBd) regions. This experiment was repeated independently, and similar results were obtained from *n* = 9 mice.

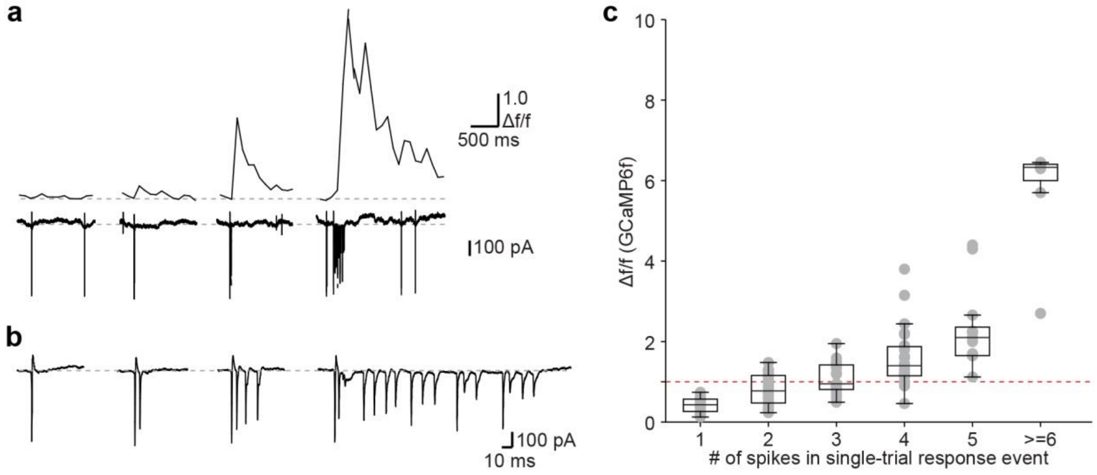

Supplementary Fig. 2 Calibrating GCaMP6f Ca^{2+} signals by loose-patch recording *in* vivo. a Example traces from simultaneous two-photon Ca^{2+} imaging and single-cell loose-patch recording in A1 L2/3 neurons in trained and behaving mice. **b** The magnified view of the loose-patch recordings is shown in panel **a**. **c** Box plots showing relations between single-trial Ca^{2+} transient amplitude versus the number of action potentials per response event. Note that the events with ≥ 6 spikes were pooled as one sample point. The red dashed line indicates the Δf/f = 1.0 threshold for identifying bursting responses (in between 3-4 spikes, corresponding to the Δf/f = 1.5 threshold for identifying bursting response by using Cal-520), and the grey dashed line indicates the detection threshold for AP-related Ca^{2+} events (Δf/f = 0.2, below which the fluorescence fluctuations are not considered spiking response events). For calculating the amplitude distributions of spike number 1 - 6, *n* = 27, 24, 19, 23, 13, 8, respectively. Boxes represent IQR, central bars indicate the median, and whiskers indicate extreme data values within Q1-1.5× IQR and Q3+1.5× IQR.

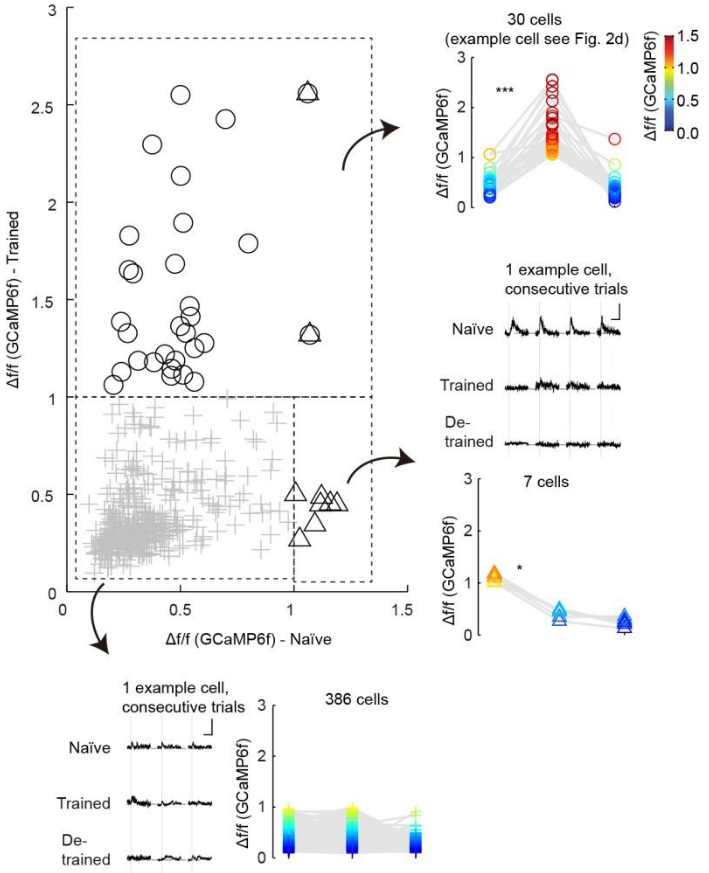

Supplementary Fig. 3 A cell-by-cell analysis of the GCaMP6f chronic Ca^{2+} imaging dataset. Upper-left 2D scatter graph: each marker (circle, triangle or cross) represents a cell (altogether 423 cells, same dataset as in Fig. 2), showing its Ca^{2+} response amplitude (averaged over available trials, minimum 3 trials) at the naïve stage (horizontal axis) and at the trained stage (vertical axis). Thirty circles show those cells with Ca^{2+} response amplitude ≥ 1.0 Δf/f at the trained stage, the amplitude difference between naïve and trained stages, two-sided Wilcoxon signed-rank test, P = 1.73e-6, unadjusted for multiple comparisons, n = 30 cells, ***$P < 0.001$; 9 triangles show those cells with Ca^{2+} response amplitude ≥ 1.0 Δf/f at the naïve stage (2 cells overlapping with circles), the amplitude difference between naïve and trained stages, two-sided Wilcoxon signed-rank test, P = 0.0156, unadjusted for multiple comparisons, n = 7 cells, *$P < 0.05$; all the other 386 cells are shown by crosses. Three arrows point to three subpanels, each showing the Ca^{2+} signals of an example cell (except for the circles, where the example cell is already shown in Fig. 2d) and the summary of all cells in the relevant group (the 2 circle-triangle overlapping cells are considered in the circle group) at naïve, trained and detrained stages. Interesting note: regardless of where one sets

the threshold for detecting high responsiveness (burst) above 0.7 Δf/f (equivalent to the median value of 2-spikes, in the main text it was set to 1.0 Δf/f, between 3 and 4 spikes), it would not change the conclusion that the cells qualifying as highly responsive at the trained stage were mostly not the cells that were already highly responsive at the naïve stage (the reverse was also true). In other words, the responsiveness between the naïve and trained stages is anti-correlational on the upper fraction, albeit correlational for the entire population.

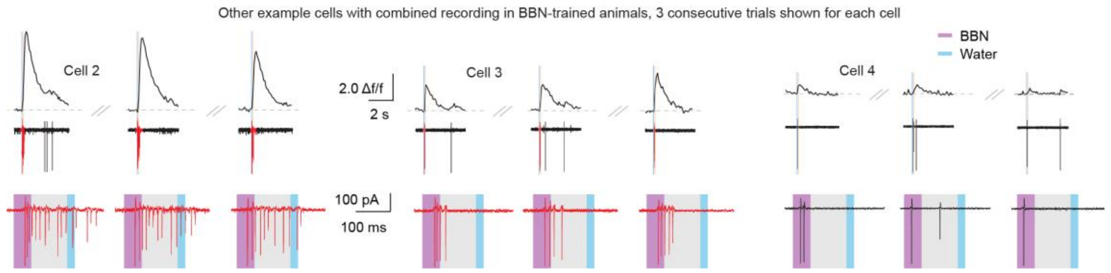

Supplementary Fig. 4 Other example cells with different numbers of spikes per event. Three Example A1 L2/3 neurons (cell label starting with 2, since Cell 1 is already shown Fig. 3b) recorded by simultaneous two-photon Ca^{2+} imaging and cell-attached electrophysiology in trained and behaving mice. Three consecutive sound-evoked response signals of 3 neurons are shown in the upper row, with the magnified view of the cell-attached recordings in the lower row. Violet and light blue vertical stripes indicate the timing of the sound stimulation and the water pumping, respectively.
