# Supplementary material for: Single-neuron representation of learned complex sounds in the auditory cortex
Source: Nat Commun. 2020 Aug 31;11:4361. doi: 10.1038/s41467-020-18142-z (PMC7459331; doi:10.1038/s41467-020-18142-z)
Supplement: Supplementary file 3 — Reporting Summary [file 41467_2020_18142_MOESM3_ESM.pdf]

nature research

Corresponding author(s): Xiaowei Chen

Last updated by author(s): July 30, 2020

Reporting Summary

Nature Research wishes to improve the reproducibility of the work that we publish. This form provides structure for consistency and transparency in reporting. For further information on Nature Research policies, seeAuthors & Referees and theEditorial Policy Checklist .

Statistics

For all statistical analyses, confirm that the following items are present in the figure legend, table legend, main text, or Methods section.

n/a	Confirmed	
☐	☒	The exact sample size (n) for each experimental group/condition, given as a discrete number and unit of measurement
☐	☒	A statement on whether measurements were taken from distinct samples or whether the same sample was measured repeatedly
☐	☒	The statistical test(s) used AND whether they are one- or two-sided<br>*Only common tests should be described solely by name; describe more complex techniques in the Methods section.*
☒	☐	A description of all covariates tested
☐	☒	A description of any assumptions or corrections, such as tests of normality and adjustment for multiple comparisons
☐	☒	A full description of the statistical parameters including central tendency (e.g. means) or other basic estimates (e.g. regression coefficient) AND variation (e.g. standard deviation) or associated estimates of uncertainty (e.g. confidence intervals)
☐	☒	For null hypothesis testing, the test statistic (e.g. F, t, r) with confidence intervals, effect sizes, degrees of freedom and P value noted<br>*Give P values as exact values whenever suitable.*
☒	☐	For Bayesian analysis, information on the choice of priors and Markov chain Monte Carlo settings
☒	☐	For hierarchical and complex designs, identification of the appropriate level for tests and full reporting of outcomes
☒	☐	Estimates of effect sizes (e.g. Cohen's d, Pearson's r), indicating how they were calculated

Our web collection on statistics for biologists contains articles on many of the points above.

Software and code

Policy information about availability of computer code

Data collection	LabVIEW 2012 (National Instruments) and Patchmaster software (HEKA Elektronik)
Data analysis	Igor Pro 5.0 (Wavemetrics) and Matlab 2014a (Mathworks)

For manuscripts utilizing custom algorithms or software that are central to the research but not yet described in published literature, software must be made available to editors/reviewers. We strongly encourage code deposition in a community repository (e.g. GitHub). See the Nature Research guidelines for submitting code & software for further information.

Data

Policy information about availability of data

All manuscripts must include a data availability statement. This statement should provide the following information, where applicable:

- Accession codes, unique identifiers, or web links for publicly available datasets
- A list of figures that have associated raw data
- A description of any restrictions on data availability

All relevant data supporting the key findings of this study are available from the corresponding author upon reasonable request. Source data underlying Figs. 1, 3–7 and Supplementary Figs. 2–3 are available as a Source data file.

Field-specific reporting

Please select the one below that is the best fit for your research. If you are not sure, read the appropriate sections before making your selection.

☒ Life sciences ☐ Behavioural & social sciences ☐ Ecological, evolutionary & environmental sciences

Life sciences study design

All studies must disclose on these points even when the disclosure is negative.

Sample size	Sample-size calculations were not performed. Samples sizes were determined based on our own previous studies (Chen et al, Nature, 2011; Li et al, Cerebral Cortex, 2017) about using behavioral, optical imaging and electrophysiological recording approaches. Samples sizes adopted in this study were sufficient for detecting robust effect.
Data exclusions	No data was excluded from analysis.
Replication	All behavioral, optical imaging and electrophysiological experiments were performed over multiple independent replicates. The study effects were all successfully replicated in the experiments.
Randomization	All samples were randomized where appropriate for data collection and processing.
Blinding	In the studies, investigators were blinded to groups for data collection and processing.

Reporting for specific materials, systems and methods

We require information from authors about some types of materials, experimental systems and methods used in many studies. Here, indicate whether each material, system or method listed is relevant to your study. If you are not sure if a list item applies to your research, read the appropriate section before selecting a response.

Materials & experimental systems

n/a	Involved in the study	
☒	☐	Antibodies
☒	☐	Eukaryotic cell lines
☒	☐	Palaeontology
☐	☒	Animals and other organisms
☒	☐	Human research participants
☒	☐	Clinical data

Methods

n/a	Involved in the study	
☒	☐	ChIP-seq
☒	☐	Flow cytometry
☒	☐	MRI-based neuroimaging

Animals and other organisms

Policy information about studies involving animals; ARRIVE guidelines recommended for reporting animal research

Laboratory animals	C57BL/6J male mice (2-3 months old) were provided by the Laboratory Animal Center at the Third Military Medical University. The mice were housed in a temperature- and humidity-controlled room on a cycle of 12 h light/dark (lights off at 19:00).
Wild animals	No wild animals were used in this study.
Field-collected samples	No field-collected samples were used in this study.
Ethics oversight	Third Military Medical University Animal Care and Use Committee

Note that full information on the approval of the study protocol must also be provided in the manuscript.
