# Supplementary material for: Single-neuron representation of learned complex sounds in the auditory cortex
Source: Nat Commun. 2020 Aug 31;11:4361. doi: 10.1038/s41467-020-18142-z (PMC7459331; doi:10.1038/s41467-020-18142-z)
Supplement: Supplementary file 4 — Description of Additional Supplementary Files [file 41467_2020_18142_MOESM4_ESM.pdf]

**Description of Additional Supplementary Files**

File Name: Supplementary Movie 1
Description: Performance of an example mouse on the sound-water association task before and after 6 sessions of training. The vertical red line denotes the sound stimulus (broadband noise, BBN).

File Name: Supplementary Movie 2
Description: An example mouse trained on 2 tones. Upper, live images of the licking behaviour in response to tone stimulus. Lower, the licking action trace. The vertical red lines indicate a 12.1 kHz tone (water from left spout, L), and the vertical green lines indicate a 2.0 kHz tone (water from right spout, R). Six consecutive trials are shown.

File Name: Supplementary Movie 3
Description: An example mouse trained on 2 chords. Upper panel, licking behaviour in response to chord stimuli. Lower panel, time course of the licking action trace. Vertical red lines indicate chord 2 (water from left spout, L), and vertical green lines indicate chord 1 (water from right spout, R). Five consecutive trials are shown. For contrast, five consecutive trials of licking behaviour in response to pure tones are shown subsequently.

File Name: Supplementary Movie 4
Description: An example of sound-evoked $Ca^{2+}$ transients in the auditory cortex of a mouse using GCaMP6f as $Ca^{2+}$ indicator before and after training. Upper panel, live images of the licking behaviour and the corresponding two-photon neuronal imaging. Lower panel, $Ca^{2+}$ signals extracted from cells in the ROIs outlined with the yellow square. Four consecutive trials are shown for each condition. The vertical red line denotes the sound stimulus (BBN).

File Name: Supplementary Movie 5
Description: An example of sound-evoked large-amplitude $Ca^{2+}$ transients in the auditory cortex of a conditioned mouse using Cal-520. Upper panel, live images of the licking behaviour and the corresponding twophoton neuronal imaging. Lower panel, licking action and $Ca^{2+}$ signals extracted from cells in the ROIs outlined with red circles. Five consecutive trials are shown. The vertical red line denotes the sound stimulus (BBN).

File Name: Supplementary Movie 6
Description: An example of sound-evoked $Ca^{2+}$ transients in the auditory cortex of a mouse using Cal-520 after 2- chord training. Upper panel, two-photon image of A1 L2/3 neurons in a trained mouse. Lower panel, $Ca^{2+}$ signals extracted from the cell in the ROIs outlined with the white square. Three consecutive trials are shown for each sound. Each vertical, light blue line denotes a sound stimulus (chord or each pure tone).
